# Ethnic variation in aortic root anatomy and prosthesis sizing in TAVI: a UK matched study

**DOI:** 10.1093/ehjimp/qyaf127

**Published:** 2025-10-17

**Authors:** Kevin Mohee, Ibrahim Antoun, Robert Ambrogetti, Shazia Hussain, Elved Roberts, Jan Kovac, Sameer Kurmani

**Affiliations:** Department of Cardiology, Glenfield General Hospital, Groby Road, Leicester LE3 9QP, UK; Department of Cardiology, Glenfield General Hospital, Groby Road, Leicester LE3 9QP, UK; Department of Cardiovascular Sciences, University of Leicester, Leicester, UK; Department of Cardiology, Glenfield General Hospital, Groby Road, Leicester LE3 9QP, UK; Department of Cardiology, Glenfield General Hospital, Groby Road, Leicester LE3 9QP, UK; Department of Cardiology, Glenfield General Hospital, Groby Road, Leicester LE3 9QP, UK; Department of Cardiology, Glenfield General Hospital, Groby Road, Leicester LE3 9QP, UK; Department of Cardiovascular Sciences, University of Leicester, Leicester, UK; Department of Cardiology, Leeds General Infirmary Hospital, Leeds, UK

**Keywords:** aortic valve stenosis, transcatheter aortic valve implantation, aortic, annular dimensions

## Abstract

**Aims:**

Ethnic variation in aortic root anatomy may influence transcatheter aortic valve implantation (TAVI) planning and outcomes. While Asian patients are reported to have smaller aortic dimensions than their Western counterparts, data for Southeast Asians in the UK are scarce.

**Methods and results:**

We retrospectively analysed all Southeast Asian patients undergoing TAVI at a large UK centre (January 2018–October 2023), matched 2:1 by age and sex to Caucasian patients. Baseline demographics, CT-derived aortic measurements, procedural details, and post-procedural outcomes were compared. Of 1230 patients undergoing TAVI, 44 Southeast Asians were matched to 84 Caucasians. Compared with Caucasians, Southeast Asians had lower BMI (25.8 ± 4.6 vs. 29.4 ± 6.5 kg/m², *P* = 0.001) and smaller annular areas (390.3 ± 89.5 vs. 469.4 ± 89.3 mm², *P* < 0.001), perimeters (71 ± 7.8 vs. 78 ± 7.6 mm, *P* < 0.001), and sinus heights (20.1 ± 3.2 vs. 22.6 ± 3.3 mm, *P* < 0.001). Indexed annular area did not differ significantly (229 ± 42.7 vs. 242 ± 41.6 mm²/m², *P* = 0.10). Median valve size was smaller in Southeast Asians (23 vs. 26 mm, *P* < 0.001). Residual aortic regurgitation (AR) post-implant was more frequent in Southeast Asians, with fewer having no AR (47.7% vs. 78.6%) and a higher proportion with mild (50.0% vs. 19.0%) or moderate AR (2.3% vs. 1.2%). Post-TAVI gradients were similar between groups.

**Conclusion:**

Southeast Asian patients in the UK undergoing TAVI have smaller annular dimensions and receive smaller prostheses compared with Caucasians, partly reflecting differences in body habitus. These findings have implications for prosthesis selection, procedural planning, and lifetime valve management.

## Graphical Abstract

**Graphical Abstract:**
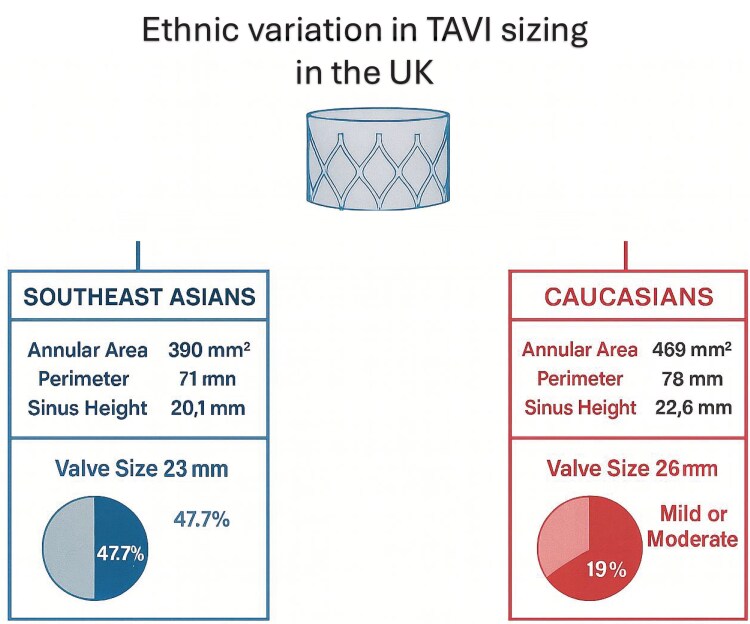

## Introduction

Aortic stenosis (AS) is one of the most common acquired forms of valvular heart disease requiring clinical intervention.^1,2^ The mortality in such patients is reported to be as high as 50% at 2 years and 97% at 5 years.^3^ Over the past 15 years, transcatheter aortic valve implantation (TAVI) has become a well-established therapeutic strategy for treating severe AS. It is increasingly applied across a broader spectrum of patient risk profiles.^4,5^ Accurate anatomical assessment of the aortic annulus and root is crucial for procedural success, as it influences prosthesis sizing, valve deployment, and ultimately, clinical outcomes.^6,7^ Multidetector computed tomography has emerged as the gold-standard imaging modality for pre-procedural assessment. While TAVI technology and procedural safety have evolved significantly, emerging evidence suggests that anatomical variations exist between ethnic groups that may affect valve selection and outcomes. Specifically, several studies have described smaller annular dimensions, narrower iliofemoral vessels, and shorter coronary heights in East and South Asian populations compared with their Western counterparts.^8–11^ While prior studies in East Asian cohorts have described smaller annular dimensions compared with Western patients, data on Southeast Asian populations, particularly within the UK, remain scarce. Given the increasing ethnic diversity of TAVI recipients, we aimed to compare aortic root and annular dimensions in Southeast Asian and Caucasian patients and explore associated procedural characteristics and outcomes.

## Methods

This is a retrospective cohort study of all patients who underwent a TAVI procedure at Glenfield Hospital in Leicester, a major tertiary cardiology service provider across the East Midlands, between January 2018 and October 2023. The data were obtained from the TAVI registry, a routinely collected database of all TAVI procedures undertaken.

Baseline demographics, including age, sex, ethnicity, cardiovascular risk factors, comorbidities, functional status, operative risk, procedural characteristics, and post-procedural outcome data, were extracted from the registry. Functional status was assessed using the Katz Index of activities of daily living, on a scale of 0–6 (from 0 = very dependent to 6 = independent), and clinical frailty using the Canadian Study of Health and Ageing clinical frailty scale.

Individual preoperative cardiac CT TAVI reports were reviewed, and patients for whom we could not access their cardiac CT reports were excluded. Four Southeast Asians and 10 Caucasians were thus excluded from the analysis.

Each Southeast Asian patient was matched with up to two Caucasian patients of the same sex and closest age. In some cases, only a single suitable Caucasian match was available. Southeast Asian ethnicity was self-reported on direct questioning by patients or their family and was defined based on birth or parental birth in India, Pakistan, Bangladesh, or Sri Lanka. The study adhered to the STROBE reporting guidelines.

### Statistical analysis

Categorical variables are presented as numbers and percentages, whereas continuous variables are presented as means ± SD, assuming normality unless otherwise specified. The statistical significance of baseline differences was determined by the χ^2^ test or *t*-test, as appropriate. Categorical data are expressed as a number with percentages. Continuous variables were compared using t-tests or Mann-Whitney U tests, as appropriate. Categorical variables were compared using the χ^2^ test or Fisher's exact test. A *P* < 0.05 was deemed statistically significant. Southeast Asian patients were matched 2:1 with Caucasian patients by age and sex, as these represent the strongest determinants of aortic annular dimensions. Additionally, variables such as BMI and comorbidities were not included in the matching process to preserve sample size; however, they were compared between groups after matching.

## Results

Between January 2018 and October 2023, 1230 patients with severe AS underwent TAVI at Glenfield Hospital. Of these 1230 patients, 48 (3.9%) were of Southeast Asian descent, and the rest were of Caucasian descent. Thus, 44 patients of Southeast Asian descent were age- and sex-matched to 84 Caucasian patients and included in the analysis.

### Baseline characteristics

The baseline characteristics of patients undergoing TAVI, stratified by either Southeast Asian or Caucasian, are shown in *Table 1*. There were significant differences in baseline patient characteristics, including larger BMIs (29.4 ± 6.5 vs. 25.8 ± 4.6, *P* = 0.001), a greater history of smoking (56.0% vs. 36.0%, *P* = 0.043), and greater independence in daily living (Katz Index median 6 vs. 5, *P* = 0.004) amongst Caucasians compared with Southeast Asians. However, there was no significant difference in the ages of the two groups (80.2 vs. 80.1 years, *P* = 0.89). Also, there was no significant difference in the proportion of males vs. females (63.6% vs. 61.4%, *P* = 0.85 Fisher’s exact test).

**Table 1:** Summary table of data from Caucasian and Southeast Asian patients undergoing TAVI

	Caucasians (*n* = 84)	Southeast Asian (*n* = 44)	*P* value
**Patient characteristics**			
Age (years)	80.2 ± 6.6	80.1 ± 6.9	0.898
Male sex (M)	56 (63.6)	27 (61.4)	0.850
BMI (kg m^−2^)	29.4 ± 6.5	25.8 ± 4.6	0.001
Diabetes	25 (29.8)	21 (47.7)	0.054
Smoking history	47 (56.0%)	16 (36.4)	0.042
Creatinine (mmol/L)	109.7 ± 44.7	124.9 ± 94.5	0.216
Katz Index (median)	6	5	0.004
Aortic valve area	0.76 ± 0.20	0.73 ± 0.19	0.508
Impaired LV	26 (31.0)	13 (29.6)	0.24
Aortic measurements			
Annulus area (mm^2^)	469.4 ± 89.3	390.3 ± 89.5	<0.001
Indexed annular area (mm^2^/m^2^)	242 ± 41.6	229 ± 42.7	0.100
Perimeter (mm)	78 ± 7.6	71 ± 7.8	<0.001
Sinus height (mm)	22.6 ± 3.3	20.1 ± 3.2	<0.001
LMS height (mm)	14.4 ± 3.5	13.6 ± 3.5	0.222
RCA height (mm)	17.1 ± 3.3	14.6 ± 2.7	<0.001
**Valve implant**			
Approach			0.089
Transfemoral	81 (96.4)	41 (93.2)	
Subclavian	2 (2.3)	1 (2.3)	
Transapical	1 (1.2)	2 (4.6)	
Valve type			
Edwards Sapien 3	29 (34.5)	18 (40.9)	
Medtronic Evolut R/PRO	40 (47.6)	19 (43.2)	
Boston Scientific Accurate Neo	12 (14.3)	4 (9.1)	
Other	3 (3.6)	3 (6.8)	
Median valve size (mm)	26 (IQR 26–29)	23 (IQR 23–26)	<0.001
Post-TAVI peak AV gradient	13.7 ± 6.3	13.7 ± 9.9	>0.05
Post-valve AR index			0.65
None	66 (78.6)	21 (47.7)	
Mild	16 (19.0)	22 (50.0)	
Moderate	1 (1.2)	1 (2.3)	
Severe	0 (0)	0 (0)	

Continuous variables are expressed as mean ± SD, and categorical variables are expressed as the median. Other data are described as the number with percentages in parentheses. *P*-values are provided from *t*-tests for continuous variables and Mann–Whitney *U* tests for categorical variables. A *P* < 0.05 was deemed statistically significant.

BMI, body mass index; LV, left ventricle; LMS, left main stem; RCA, right coronary artery; TF, transfemoral; SC, subclavian; TA, transapical; AV, aortic valve; na, not applicable.

### CT and procedural characteristics

**Figure 1:**
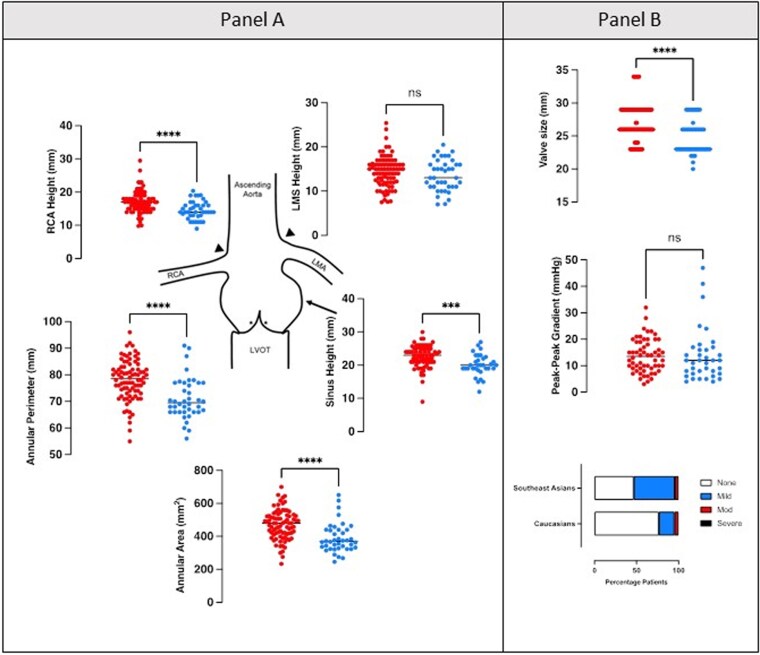
Aortic dimensions and valve implantation in Southeast Asian and Caucasian patients. Panel *A* depicts annular measurements for Caucasian (shown in red) and Southeast Asian patients (shown in blue). Graphs demonstrate individual values with the group mean (black line). Panel *B* depicts valve characteristics. The top graph depicts valve sizes (regardless of manufacturer) in both patient groups. The middle graph shows the peak-to-peak pullback gradient. The bar chart at the bottom demonstrates the proportion of patients in each group with none, mild, moderate, and severe AR by echocardiography. **** denotes *P* < 0.05. LVOT, left ventricle outflow tract; RCA, right coronary artery; LMA, left main coronary artery.

Concerning aortic anatomy, Caucasians had larger annular areas (469.4 ± 89.3 vs. 390.3 ± 89.5 mm^2^, *P* < 0.001), perimeters (78 ± 7.6 vs. 71 ± 7.8 mm, *P* < 0.001), and sinus heights (22.6 ± 3.3 vs. 20.1 ± 3.2 mm, *P* < 0.001). When adjusted for body surface area, there was no overall significant difference in annular area (242 ± 41.6 vs. 229 ± 42.7 mm^2^/m^2^, *P* = 0.100). Overall, there were significantly smaller median valve sizes implanted in the Southeast Asian population compared with the Caucasian population (26 vs. 23 mm, *P* < 0.001). Both approaches used and the type of valve implanted in each patient group were comparable; however, the median valve size was larger in the Caucasian population (26 vs. 23 mm, *P* < 0.001). There was no significant difference in the post-implant peak-to-peak AV gradient (13.7 ± 6.3 vs. 13.7 ± 9.9 mmHg, *P* = 0.67). However, there was a greater proportion of patients with no residual aortic regurgitation (AR) after implant in the Caucasian population as compared with the Southeast Asian population (78.6% vs. 47.7%), as shown in *Figure 1*.

## Discussion

This retrospective cohort study demonstrates that patients of Southeast Asian descent undergoing TAVI have significantly smaller annular areas, perimeters, and sinus heights than age- and sex-matched Caucasian patients. These findings are broadly consistent with prior research indicating that patients of Southeast Asian ancestry tend to have smaller aortic root dimensions, even after indexing for body surface area.^8–12^ However, this is the first study to describe these anatomical differences in a UK Southeast Asian population, a demographic that forms a substantial part of the ethnic minority community nationally.^13^ A notable finding in our study is the smaller valve sizes implanted in Southeast Asian patients compared with Caucasians, with a median difference of 3 mm (23 vs. 26 mm, *P* < 0.001). While this difference is partly explained by lower BMI and a smaller indexed annular area, the persistently smaller raw measurements highlight the importance of considering ethnic variation in procedural planning and valve sizing strategies. These anatomical differences highlight the potential value of next-generation prostheses designed for smaller annuli, including devices with lower profile delivery systems and improved sealing skirts. Similarly, procedural strategies such as intentional oversizing within safe limits, alternative access routes, or the use of balloon-expandable valves in specific anatomies may mitigate residual AR risk.

Inadequate sizing could increase the risk of paravalvular regurgitation, annular rupture, or conduction abnormalities.^7,14,15^ Interestingly, the incidence of residual AR post-TAVI was higher in Southeast Asian patients, despite similar peak-to-peak gradients and access approaches. While the differences in AR may reflect valve selection or deployment dynamics in smaller annuli, further work is needed to confirm this association. Comparable studies in Korean, Japanese, and Chinese cohorts have similarly reported smaller valve sizes but have not consistently described a higher rate of post-TAVI AR.^9–11^ Conduction abnormalities such as new left bundle branch block and the need for permanent pacemaker implantation are closely related to prosthesis design, annular dimensions, and implantation depth. While our dataset did not reliably capture conduction outcomes, future work in Southeast Asian populations should examine this aspect, given their smaller annuli and potentially heightened risk.

Furthermore, the higher prevalence of residual AR among Southeast Asian patients may reflect not only smaller annular dimensions but also interactions with prosthesis type. Balloon-expandable valves typically achieve more complete circular expansion and lower rates of paravalvular regurgitation. In contrast, self-expanding devices, although advantageous for smaller annuli and lower gradients, are more susceptible to incomplete sealing, particularly in anatomies with greater eccentricity or calcification. Although valve type distribution was broadly similar between groups in our study, the interplay between annular anatomy and prosthesis mechanics may contribute to the observed differences in residual AR. Future studies incorporating detailed CT-derived calcium mapping and annular geometry analysis are warranted. Previous studies have shown that prosthesis–patient mismatch and incomplete sealing are more likely in anatomies with smaller, more elliptical annuli.^7,14,15^ Detailed analysis of annular eccentricity and calcium burden in this population may help refine device selection and sizing. The underrepresentation of Southeast Asians in TAVI cohorts, despite their substantial presence in the UK population, underscores the need for targeted outreach and equitable referral pathways. Identifying anatomical and clinical features earlier in the disease course may enable timely intervention and improve procedural outcomes.

Our findings also corroborate previous UK registry data highlighting the disproportionate underrepresentation of ethnic minorities in TAVI cohorts, with Southeast Asians comprising just 3.9% of patients undergoing the procedure over 5 years, despite forming nearly 9% of the UK population.^13,16^ The disparity in TAVI procedures between ethnic groups may partly reflect a different distribution of ethnicities across various age groups in the UK. However, prior studies suggest that disparities are likely multifactorial, involving socioeconomic factors, referral bias, differences in disease phenotype, and patient choice.^15–17^ The significantly higher Katz independence index among Caucasians in our cohort may also suggest that functional status or perceived surgical candidacy could influence TAVI referral decisions. This study contributes valuable real-world evidence from a large-volume UK centre and may inform future TAVI device design, pre-procedural planning algorithms, and population-specific guidelines. The use of AI-based valve sizing and annular assessment tools incorporating ethnicity and body habitus may further refine precision valve therapy in diverse populations.^18–20^ Although multivariable modelling would be desirable to adjust for valve type, size, and annular characteristics, the relatively small sample size in this study precludes robust regression analysis without the risk of model overfitting. Instead, we performed an exploratory descriptive assessment, which indicated that residual AR was more common among Southeast Asian patients receiving self-expanding valves and smaller prostheses. This supports the hypothesis that annular size, prosthesis interaction, and sealing dynamics may underlie the observed differences. Larger multicentre studies are warranted to confirm these associations.

## Limitations

Strengths of this study include its real-world nature, analysis from a high-volume UK TAVI centre, and direct age- and sex-matched comparison between Southeast Asian and Caucasian patients using contemporary CT-derived measurements. To our knowledge, this is the first UK study specifically addressing Southeast Asian anatomical differences in TAVI recipients.

However, several limitations must be acknowledged. This is a single-centre retrospective study with a relatively small Southeast Asian cohort, which may limit generalizability. Four subjects from the Southeast Asian group were excluded due to an inability to access complete CT TAVI results, potentially impacting results. Matching was performed only for age and sex, and residual confounding may remain. Matching was limited to age and sex, which are key anatomical determinants. While BMI, valvular characteristics, prosthesis implantation techniques, and comorbidities were excluded to avoid excessive reduction in sample size, this may still allow for residual confounding. We were unable to analyse conduction abnormalities (new bundle branch block or pacemaker requirement) as these outcomes were not systematically recorded, which is a limitation given their known relationship to annular anatomy and valve type. Ethnicity classification relied on self-report, which may be subject to misclassification. CT-derived annular measurements were pre-procedural only; post-implant imaging was not available to evaluate true prosthesis–patient mismatch or valve expansion. Finally, procedural decision-making (valve type, oversizing strategy) was operator-dependent, which may influence outcomes such as residual AR.

## Conclusion

In this UK cohort, Southeast Asian patients undergoing TAVI had significantly smaller annular and root dimensions than matched Caucasian patients, leading to smaller valve implants. Although partly attributable to body habitus, these differences may affect prosthesis selection, procedural strategies, and long-term valve durability. Tailoring device choice and deployment techniques to account for ethnic anatomical variation may optimize outcomes and inform future TAVI guidelines.

## Data Availability

Data relating to this study are available upon reasonable request from the corresponding author.

## Lead author biography

Dr Ibrahim Antoun is UK licensed physician and a member of the Royal College of Physicians in the United Kingdom in 2021. Completed his PhD in Electrophysiology in the University of Leicester in 2023. Currently works as a Cardiology trainee in East Midlands deanery and an honorary research fellow at the University of Leicester.
